# Author Correction: Herbivores rescue diversity in warming tundra by modulating trait-dependent species losses and gains

**DOI:** 10.1038/s41467-017-02129-4

**Published:** 2017-12-20

**Authors:** Elina Kaarlejärvi, Anu Eskelinen, Johan Olofsson

**Affiliations:** 1 Climate Impacts Research Centre (CIRC), Department of Ecology and Environmental Science, Umeå University, SE-981 07, Abisko, Sweden; 2 Department of Biology, Vrije Universiteit Brussel (VUB), Pleinlaan 2, 1050 Brussels, Belgium; 3 Department of Physiological Diversity, Helmholtz Center for Environmental Research—UFZ, Permoserstr. 15, D-04318 Leipzig, Germany; 4 German Centre for Integrative Biodiversity Research (iDiv) Halle-Jena-Leipzig, Deutscher Platz 5e, D-04103 Leipzig, Germany; 5 Department of Ecology, University of Oulu, P.O. Box 3000, FI-90014 Oulu, Finland

*Nature Communications* **8**:419 10.1038/s41467-017-00554-z; Article published online: 4 Sep 2017

The original version of this Article contained errors in Figure 2. The dark green symbols on the scatter plot were light green, and vice versa. These errors have now been corrected in the PDF and HTML versions of the Article.

