# Supplementary material for: Herbivores rescue diversity in warming tundra by modulating trait-dependent species losses and gains
Source: Nat Commun. 2017 Sep 4;8:419. doi: 10.1038/s41467-017-00554-z (PMC5583392; doi:10.1038/s41467-017-00554-z)
Supplement: Supplementary file 1 — Supplementary Information [file 41467_2017_554_MOESM1_ESM.pdf]

Type of file: pdf

Title of file for HTML: Supplementary Information

Description: Supplementary Figures, Supplementary Tables, and Supplementary References

----

Type of file: pdf

Title of file for HTML: Peer Review File

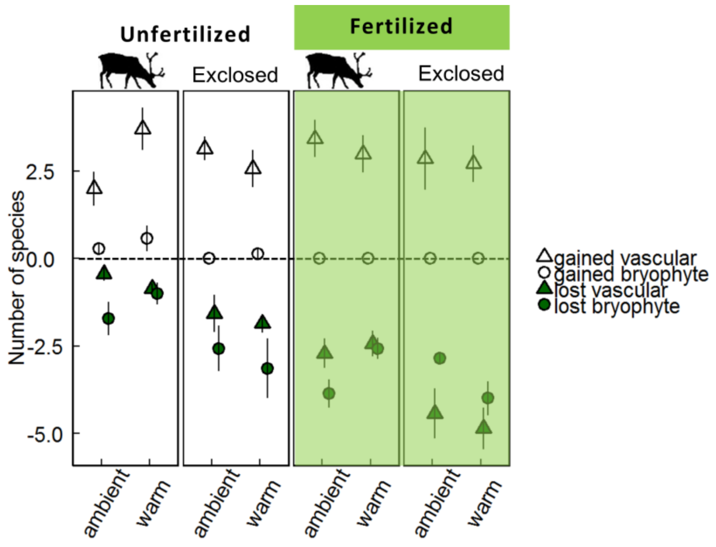

Supplementary Figure 1. Number of lost and gained vascular plants and bryophytes. Change in number of species (means and SE, n=7 for each point) under warming, herbivore exclusion and fertilization treatments between 2009 and 2014. Vascular plant and bryophyte richness at the start of the experiment was 17.9 ± 0.46 and 3.7 ± 0.19 species in 25 × 50 cm^2 quadrats (mean±SE, n=56), respectively.

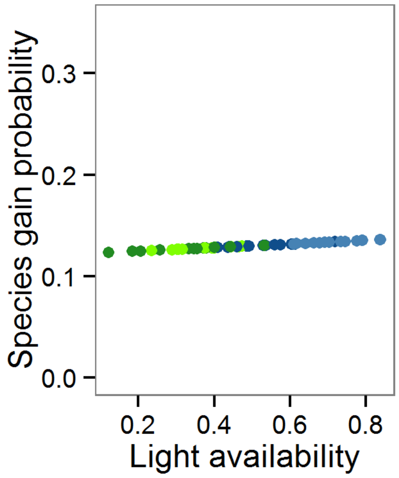

Supplementary Figure 2. Impact of light availability on species gain probability. Relationship between light availability (measured as proportion of ambient PAR penetrating through vegetation) and species gain probability in fertilization and herbivore exclusion treatments. Fertilized plots are shown in green, unfertilized in blue. Herbivore exclosures are indicated with dark color, grazed plots in light color.

Supplementary Table 1. Impacts of warming, grazing and fertilization on species richness. Results of ANOVA of effects of treatments and their interactions on change in species richness of all plants (vascular plants + bryophytes), vascular plants and bryophytes between 2009 and 2014. Each treatment combination has 1 numerator and 48 denominator degrees of freedom. P-values <0.05 are considered significant and indicated in bold.

	All plants		Vascular plants		Bryophytes	
	F	*P*	*F*	*P*	*F*	*P*
Warming (W)	0.03	0.870	0.02	0.886	0.24	0.625
Herbivore exclusion (E)	18.74	**<0.001**	12.95	**<0.001**	8.14	**0.006**
Fertilization (F)	33.11	**<0.001**	21.22	**<0.001**	16.27	**<0.001**
W × E	6.08	**0.017**	1.68	0.567	7.06	**0.012**
W × F	0.36	0.545	0.33	0.201	0.09	0.769
E × F	0.03	0.870	2.07	0.156	5.12	**0.028**
W × E × F	0.08	0.786	0.75	0.392	0.47	0.494

Supplementary Table 2. Impacts of warming, grazing and fertilization on species gains and losses. Results of ANOVA of effects of treatments and their interactions on numbers of lost and gained species of all plants, vascular plants and bryophytes between 2009 and 2014. Number of gained bryophytes were virtually zero and thus could not be modeled. Each treatment combination has 1 numerator and 48 denominator degrees of freedom. Transformed response variables are indicated in footnotes. P-values <0.05 are considered significant and indicated in bold and marginally significant values (P<0.1) are indicated in bold italics.

	Lost plants		Gained plants[1]		Lost vascular plants[2]		Gained vascular plants		Lost bryophytes	
	F	*P*	*F*	*P*	*F*	*P*	*F*	*P*	*F*	*P*
Warming (W)	0.07	0.789	0.40	0.530	0.16	0.694	0.13	0.726	0.04	0.840
Herbivore exclusion (E)	21.02	**<0.001**	0.80	0.375	18.75	**<0.001**	0.28	0.599	5.92	**0.019**
Fertilization (F)	47.29	**<0.001**	0.08	0.777	35.89	**<0.001**	0.13	0.726	11.88	**0.001**
W × E	4.09	**0.049**	1.31	0.258	0.16	0.691	1.53	0.223	6.95	**0.011**
W × F	0.07	0.789	1.12	0.295	0.01	0.940	1.12	0.295	0.00	1.000
E × F	0.07	0.789	0.14	0.714	5.89	**0.019**	0.28	0.599	3.33	***0.074***
W × E × F	0.89	0.350	3.02	***0.089***	0.32	0.573	2.53	0.119	0.66	0.421

Transformations: [1]square root, [2]log10

Supplementary Table 3. Results of logistic regression analysis of tested factors' effects on probabilities of species losses. Summary of logistic regression models predicting effects of species' initial abundance (2009), light availability (proportion of PAR penetrating vegetation) and functional traits on probabilities of species' loss under indicated treatment combinations. Models including functional traits were simplified (see Methods). Number of replicates slightly varies due to missing trait values for some species.

	**Estimate**	**z-value**	***P***
**Initial abundance (n=1000)**			
Intercept	-1.486	-9.235	<0.001
Abundance 2009	-0.076	-4.238	<0.001
**Proportion PAR (n=1000)**			
Intercept	-0.170	-0.632	0.528
Proportion PAR	-3.773	-6.226	<0.001
**Proportion PAR and initial abundance (n=1000)**			
Intercept	-0.042	-0.135	0.893
PAR	-2.746	-3.779	<0.001
Abundance 2009	0.021	0.901	0.368
PAR × Abundance 2009	-0.258	-3.026	0.002

**Height (n=1000)**			
Intercept	1.084	2.875	0.004
Warming (W)	-0.132	-0.616	0.538
Exclosure (E)	0.432	0.706	0.481
Fertilization (F)	-0.849	-1.390	0.165
Abundance 2009	-0.031	-1.979	0.048
Height	-0.110	-5.117	<0.001
E × Height	-0.048	-1.209	0.227
F × Height	0.021	0.559	0.576
E × F	-1.955	-2.064	0.039
E × Abundance 2009	-0.183	-2.815	0.005
F × Abundance 2009	-0.301	-2.915	0.004
E × F × Height	0.117	1.911	0.056
**SLA (n=999)**			
Intercept	1.041	2.473	0.013
Warming (W)	0.110	-0.541	0.589
Exclosure (E)	0.240	-0.874	0.382
Fertilization (F)	0.679	-2.093	0.036
Abundance 2009	0.032	-2.116	0.034
SLA	0.080	-4.514	<0.001
E × Abundance 2009	0.140	-2.708	0.007
F × Abundance 2009	0.267	-2.749	0.006
**log(C:P) (n=955)**			
Intercept	-14.367	-5.160	<0.001
Warming (W)	-0.210	-0.951	0.342
Exclosure (E)	-0.091	-0.304	0.761
Fertilization (F)	8.648	1.820	0.069
Abundance 2009	-0.030	-1.994	0.045
log (CP)	2.407	4.940	<0.001
E × Abundance 2009	-0.142	-2.667	0.008
F × Abundance 2009	-0.255	-2.346	0.019
F × log(CP)	-1.659	-1.989	0.047
**C:N (n=997)**			
Intercept	-3.335	-5.459	<0.001
Warming (W)	1.464	1.969	0.049
Exclosure (E)	-0.252	-0.913	0.361
Fertilization (F)	1.263	1.490	0.136
Abundance 2009	-0.039	-2.520	0.012
CN	0.124	4.743	<0.001
E × Abundance 2009	-0.141	-2.681	0.007
F × Abundance 2009	-0.260	-2.645	0.008
W × CN	-0.068	-2.135	0.033
E × CN	0.063	1.753	0.080
F × CN	-0.088	-2.408	0.016
**Condensed tannins (n=978)**			
Intercept	-0.829	-4.034	<0.001
Abundance 2009	-0.018	-1.393	0.164
Warming (W)	-0.186	-0.906	0.365
Exclosure (E)	-0.242	-0.884	0.377
Fertilization (F)	-0.505	-1.547	0.122

Tannins	0.006	1.931	0.053
E × Abundance 2009	-0.123	-2.459	0.014
F × Abundance 2009	-0.274	-2.811	0.005
**log(Total phenolics (n=953)**			
Intercept	-1.829	-3.484	<0.001
Abundance 2009	-0.015	-1.284	0.199
Exclosure (E)	-0.117	-0.414	0.679
Fertilization (F)	-0.735	-2.038	0.042
Warming (W)	-0.283	-1.307	0.191
Phenols	0.296	2.181	0.029
E × Abundance 2009	-0.125	-2.551	0.011
F × Abundance 2009	-0.269	-2.462	0.014

Supplementary Table 4. Results of logistic regression analysis of tested factors' effects on probabilities of species gains. Summary of simplified logistic regression models predicting effects of functional traits on probabilities of species gains under indicated treatment combinations.

	Estimate	z-value	*P*
**Community biomass in 2014 (n=1257)**			
Intercept	-0.709	-0.375	0.708
log(Community biomass)	-0.169	-0.632	0.527
**Height (n=1257)**			
Intercept	-2.241	-6.661	<0.001
Warming (W)	0.275	0.579	0.563
Exclosure (E)	0.544	1.184	0.236
Fertilization (F)	0.434	0.937	0.349
Height	0.008	0.938	0.349
W × E	-0.866	-1.302	0.193
W × F	-0.271	-0.400	0.689
E × F	-1.558	-2.237	0.025
W × Height	-0.006	-0.479	0.632
E × Height	-0.009	-0.685	0.494
F × Height	-0.007	-0.572	0.568
W × E × F	2.393	2.463	0.014
W × E × Height	0.020	1.104	0.270
W × F × Height	0.001	0.050	0.960
E × F × Height	0.023	1.239	0.216
W × E × F × Height	-0.045	-1.649	0.099
**SLA (n=1257)**			
Intercept	-2.613	-4.724	<0.001
Warming (W)	0.119	0.348	0.728
Exclosure (E)	-1.117	-1.401	0.161
Fertilization (F)	-0.642	-0.809	0.419
SLA	0.030	1.188	0.235
W × E	-0.307	-0.644	0.520
W × F	-0.273	-0.559	0.576
E × F	0.940	0.815	0.415
E × SLA	0.066	1.943	0.052
F × SLA	0.043	1.239	0.216
W × E × F	1.240	1.796	0.073
E × F × SLA	-0.087	-1.775	0.076
**C:N (n=1203)**			
Intercept	-3.007	-4.137	<0.001
Warming (W)	0.183	0.525	0.600
Exclosure (E)	1.708	1.836	0.066
Fertilization (F)	1.701	1.795	0.073

CN	0.045	1.441	0.150
W × E	-0.405	-0.853	0.394
W × F	-0.299	-0.610	0.542
E × F	-3.086	-2.354	0.019
E × CN	-0.063	-1.513	0.130
F × CN	-0.067	-1.561	0.119
W × E × F	1.316	1.916	0.055
E × F × CN	0.100	1.697	0.090
**Condensed tannins (n=995)**			
Intercept	-1.825	-6.316	<0.001
Warming (W)	0.626	1.536	0.125
Exclosure (E)	0.938	2.401	0.016
Fertilization (F)	0.658	1.644	0.100
Tannins	-0.005	-0.569	0.569
W × E	-1.100	-1.980	0.048
W × F	-1.094	-1.883	0.060
E × F	-1.591	-2.776	0.006
W × Tannins	-0.040	-1.702	0.089
E × Tannins	-0.055	-1.934	0.053
F × Tannins	-0.025	-1.518	0.129
W × E × F	2.293	2.849	0.004
W × E × Tannins	0.073	1.925	0.054
W × F × Tannins	0.066	2.297	0.022
E × F × Tannins	0.062	1.760	0.079
W × E × F × Tannins	-0.100	-2.184	0.029
**Total phenolics (n=964)**			
Intercept	-1.678	-7.663	<0.001
Warming (W)	0.227	1.229	0.219
Exclosure (E)	0.055	0.297	0.766
Fertilization (F)	0.082	0.445	0.657
Phenolics	-0.005	-2.308	0.021

Supplementary Table 5. Species and groups of taxa with their functional traits. Functional traits for the dominant species (underlined) of each group of taxa were used as traits for the whole group.

Species or group taxon	Height (cm)	SLA (mm^2/mg)	C:N	C:P	Condensed tannins (mg/g)	Total phenolics (mg/g)	Data source
Achillea millefolium	43.1	10.2	15	193.6	NA	NA	TRY, Garnier *et al.*[1]
Alchemilla sp. + *alpina*	23.8	21.3	23.8	317.2	3.1	142	own collection 2014
Antennaria dioica + *alpina*	10.5	14.3	30.8	358.9	1.0	54.7	own collection 2014
Anthoxantum odoratum + *Poa alpigena* + *Hierochloë hirta*	24.6	27.4	17.4	317.5	1.0	10	own collection 2014
Astragalus alpinus + *frigidus*	9.8	16.2	10.9	169.7	1.0	19.8	own collection 2014
Bistorta vivipara	15.3	14.5	17.9	237.3	93.2	90.5	own collection 2014
Bothrychium lunaria	5	17.7	NA	NA	NA	NA	TRY, Fitter *et al.*[2]
Calamagrostis lapponica	91.6	12.2	20.6	NA	NA	NA	own collection 2010
Carex bigelowii + *vaginata* + *norwegica*	14.4	18.4	18.4	309	1.0	18.6	own collection 2014
Cerastium alpinum	8.8	20.2	15.2	NA	10.	NA	own collection 2010
Coeloglossum viride	NA	NA	NA	NA	NA	NA	
Deschampsia cespitosa	82.6	11.6	26.8	409.6	1.0	10.7	own collection 2014
Deschampsia flexuosa + *Festuca ovina*	40.1	13.6	31	341.2	1.0	13.6	own collection 2014
Equisetum pratense + *sqirpoides*	21.7	16.5	23.6	425.7	1.0	19.0	own collection 2014
Erigeron uniflorum	10.7	23.9	19.6	NA	NA	NA	TRY, Pierce *et al.*[3]
Euphrasia frigida	8.1	14.6	12	147	2.6	31.9	own collection 2014
Gentiana nivalis	8.9	28.5	23.5	432.9	1.0	25.9	own collection 2014
Gerastium sylvaticum	43.6	23.8	18.9	211.9	1.0	165	own collection 2014
Gnaphalium supinum	4.6	35.8	18.4	250.6	1.0	47.2	own collection 2014
Hieracium alpinum + *Leontodon autumnalis*	19.1	20.8	22.1	253	1.0	33.1	own collection 2014
Linnea borealis	6.3	15.4	44.6	350.9	1.0	23.1	own collection 2014
Luzula multiflora + *spicata*	15.9	23.9	18.8	185.4	1.0	25.9	own collection 2014
Nardus stricta	17	NA	NA	NA	NA	NA	own collection 2014
Oxyria digyna	17.4	19.6	16	216.3	14.9	28.6	own collection 2014
Poa alpina + *Phleum alpinum*	25.2	21	26.2	279.1	1.0	17.1	own collection 2014
Potentilla cranzii	16.2	13	21.7	314	5.8	86.6	own collection 2014
Pyrola minor	11.5	15.4	30.4	362.4	60.8	99.3	own collection 2014
Ranunculus acris	27.2	22.5	19.6	232	1.0	27.9	own collection 2014
Rumex lapponum	43.5	23.6	18.4	301.8	56.8	55.3	own collection 2014
Salix hastata + *phylicipholia*	13.4	13.2	27.7	NA	NA	NA	own collection 2010
Salix herbarea	3.7	15.8	23.8	360.2	125.1	128	own collection 2014
Salix reticulata	3.5	10.5	22.3	379.2	105.0	83.8	own collection 2014
Saussurea alpina	24.8	23.6	22.3	395	1.0	22.8	own collection 2014

Selaginella selaginoides	3.5	NA	24.8	363.5	1.0	17.2	own collection 2014
Sibbaldia procumbens	4.4	17.9	17.9	195.6	43.5	188	own collection 2014
Solidago virgaurea	31.2	31.8	16.8	107.7	1.0	20.4	own collection 2014
Taraxacum spp.	25.9	27.5	15.8	250.1	1.0	29.6	own collection 2014
Thalictrum alpinum	6.4	15.4	22.4	417.5	1.0	21.5	own collection 2014
Trientalis europea	6.8	30.8	31.9	292.4	41.1	55.6	own collection 2014
Trisetum spicatum	15.3	12.8	4.7	NA	NA	NA	TRY, Spasojevic et al[4]
Trollius europeus	66.5	25.1	22.7	222.7	1.0	32.6	own collection 2014
Vaccinium myrtillus	9.4	20.5	30.3	416.9	75.4	103	own collection 2014
Vaccinium uliginosum	12.4	15	25.8	207.4	52.0	81.1	own collection 2014
Vaccinium vitis-idaea	9.2	11.3	39.1	433.3	8.5	33.3	own collection 2014
Veronica alpina	7.3	15.5	26.7	436.2	3.3	41.0	own collection 2014
Viola biflora	6.8	36.7	13.8	178.9	1.0	11.7	own collection 2014

Supplementary References

1. Garnier, E. *et al.* Assessing the effects of land-use change on plant traits, communities and ecosystem functioning in grasslands: a standardized methodology and lessons from an application to 11 european sites. *Ann. Bot.* **99,** 967–985 (2007).
2. Fitter, A. H. & Peat, H. J. The Ecological Flora Database. *J. Ecol.* **82,** 415–425 (1994).
3. Pierce, S., Luzzaro, A., Caccianiga, M., Ceriani, R. M. & Cerabolini, B. Disturbance is the principal α-scale filter determining niche differentiation, coexistence and biodiversity in an alpine community. *J. Ecol.* **95,** 698–706 (2007).
4. Spasojevic, M. J. & Suding, K. N. Inferring community assembly mechanisms from functional diversity patterns: the importance of multiple assembly processes. *J. Ecol.* **100,** 652–661 (2012).
